# Trends in childhood obesity for upper tier local authorities in England between 2007/08 and 2023/24: a latent trajectory analysis

**DOI:** 10.1093/pubmed/fdaf103

**Published:** 2025-08-19

**Authors:** John Rahilly, Mario Cortina-Borja, Oliver Mytton

**Affiliations:** Population, Policy and Practice Department, UCL Great Ormond Street Institute of Child Health, 30 Guildford St., London WC1N 1EH, UK; Population, Policy and Practice Department, UCL Great Ormond Street Institute of Child Health, 30 Guildford St., London WC1N 1EH, UK; Population, Policy and Practice Department, UCL Great Ormond Street Institute of Child Health, 30 Guildford St., London WC1N 1EH, UK

**Keywords:** children, models, obesity

## Abstract

**Background:**

Childhood obesity is a major public health issue, with significant variation across England. Understanding the causes of this variation can offer insights to improve practice. We aimed to identify areas diverging from national trends using data from 150 Local Authorities (LAs) in the National Child Measurement Programme (2007/08–2023/24).

**Methods:**

Growth mixture models identified classes with distinct trajectory profiles. Logistic regression tested whether deprivation (Indices of Multiple Deprivation/Income Deprivation Affecting Children Index) and ethnicity predicted class assignment.

**Results:**

For both Reception and Year 6, two trajectory classes emerged. Reception Class I: ‘Moderate & Stable’ (132/150, 88%); Class II: ‘High & Declining’ (18/150, 12%). Year 6 Class I: ‘Moderate & Rapidly Increasing’ (135/150, 90%); Class II: ‘High and Gradually Increasing’ (15/150, 10%). Assignment to Class II was associated with higher deprivation; a higher proportion of children from ethnic minority groups; and relative reductions in deprivation and shifts in school ethnic composition.

**Conclusion:**

A small number of LAs, predominantly in South-East England, have a less adverse trend in child obesity prevalence compared to the majority. Assignment to this group was associated with higher deprivation; a higher proportion of children from ethnic minority groups; and reducing deprivation and changing ethnicity over time.

## Introduction

The numbers of children living with obesity represent a significant public health problem.^1^ In England prevalences are high,^2^ but stable for children aged 4–5 years (9.6% in 2007/08 and 2023/24), and increasing for children aged 10–11 years (18.3% and 22.1%, respectively).^3^ These overall figures mask important health inequalities, with a 6.9 percentage points gap between the most and least deprived deciles for children aged 4–5 years and 16.1 for children aged 10–11 years in 2023/24, having increased since 2007/08.^4^ Residual disparities in prevalence are also recorded between different ethnic groups.^5^

There are also regional differences. In 2023/24 the lowest prevalences were recorded in the South-East (8.6% and 19.2%) and South-West (8.8% and 19.1%), whilst the highest were in the North-East (10.8% and 24.5%) and West Midlands (10.9% and 24.4%).^6^ Deprivation and ethnicity will explain some of these differences.^7^

In England there has been significant national effort to tackle childhood obesity: three national plans between 2016 and 2020;^8–10^ the introduction of ‘population-level’ measures including a levy on added sugar soft drinks;^11^ and restrictions around food marketing^12^; investment in child weight management services;^13^ and new tools for local government to deliver systems change.^14^ Whilst often overlooked in national policy making, local government has a statutory duty to improve population health and is responsible for the delivery of public health programmes.^15^ Moreover, it is responsible for many non-health services and functions (e.g. parks, education, planning and trading standards) that influence the healthiness of the environment for children’s growth. Some local authorities have implemented interventions (e.g. advertising and takeaway restrictions) that are effective in limiting negative environmental influences.^16^^,^^17^ As well as differences in socio-economic factors, there is likely considerable variation in approaches to tackling childhood obesity between areas. As in other areas of health practice, the identification of ‘outliers’ can be informative, by subsequently helping to identify modifiable factors to improve outcomes.^7^^,^^18–20^

In England, annually since 2006/07 heights and weights of children in the first (i.e. Reception, aged 4–5 years) and last years (i.e. Year 6, aged 10–11 years) attending state-funded primary schools have been measured for the National Child Measurement Programme (*NCMP*). As part of this surveillance programme, children whose body mass index z-score (z-BMI) score (adjusted for age and sex) is at or exceeds the relative value for the 95^th^ percentile of the British Growth Reference 1990,^21^ are classified as living with obesity. Based on this, prevalence of obesity is reported annually for each Local Authoritie (LA), presenting an opportunity to explore differences between areas over time.

To date limited work has been undertaken to explore these differences. That which already exists is now relatively old, having largely occurred before the period of national and local policy innovation.^7^^,^^18^^,^^22^ It is timely to revisit the question of whether outlier LAs exist. We chose to identify if groups of authorities diverge from others, rather than focus on identifying single outlier LAs, because of the risk of over-interpreting single outliers and to improve generalizability. To do this we used a latent trajectory approach, building on previous work*.*^7^

## Methods

We fitted latent growth mixture models (LGMM), to identify distinct groups based upon longitudinal data. Once distinct groups were identified, we tested whether socio-demographic data, or changes over time predicted group membership.

### Childhood obesity data

We focused upon obesity only (≥95th centile), to be consistent with prior academic literature and public policy;^7^^,^^23^^,^^24^ and used annual population obesity prevalence data for Reception and Year 6 (2007/08–2023/24) from the NCMP, for 152 upper tier LAs in England, which existed at the start of the research period. *City of London* and *Isles of Scilly* are small areas and their data is aggregated into their nearest geographic neighbours (*Hackney* and *Cornwall*^25^), resulting in a final sample of 150 LAs. We excluded data from 2019/20 and 2020/21 due to sampling and statistical uncertainty associated with restrictions to measurement during the COVID-19 pandemic. All data represent both sexes combined.

### Socio-demographic data

We used two local-area measures of deprivation: Indices of Multiple Deprivation (IMD) and Income Deprivation Affecting Children Index (IDACI), obtained for 2010,^26^ 2015^27^ and 2019^28^ at Lower Super Output Area, then aggregated to derive population-weighted averages for each upper tier LA, based upon total population.

Data on ethnicity for all primary school-age children was taken from the *Schools, Pupils And Their Characteristics* annual census from 2009/10 to 2023/24.^29^

We aggregated the 18 Office for National Statistics’ ethnic group categories into five groups (White, Black, South Asian, Other Asian, or Other groups), to match previous work on ethnicity, *z*-BMI and adiposity.^30^ We additionally accounted for non-disclosed ethnicity with a sixth group (‘Unclassified’).

Due to differences in methodological approaches between years, it is not appropriate to compare deprivation scores between time points.^31^ Average IMD and IDACI scores were assigned a relative rank at each time point and change in deprivation over time was conceptualized as two inverted continuous variables reflecting the difference between rank in 2010 and 2019. Change in ethnicity reflected a continuous absolute change in percentage of primary school population between 2009/10 and 2023/24.

### Statistical methods

Analyses were undertaken in R.^32^^,^^33^ We used an unadjusted three-stage approach, in which (a) an initial latent class model was fitted based upon trends in obesity prevalence; (b) posterior probabilities were estimated for class assignment; and (c) associations were tested between class assignment and predictor variables using logistic regression.^34^

LA obesity prevalence was treated as a continuous, normally distributed variable, with time coded as a regularly spaced single increasing integer (e.g. 2007/08 = 1,..., 2023/24 = 15), excluding 2019/20 and 2020/21. We fitted linear LGMMs initially with fixed intercept and slope, then allowing random effects and assessed model fit statistics. Linear LGMMs allowing for random intercept and slope were fitted sequentially increasing from an initial single-class model. Model fit statistics were interpreted after each iteration to assess optimal parameters.^7^^,^^35^ Model fit statistics included Akaike information criterion (AIC), adjusted Bayesian information criterion (BIC) and entropy (the precision with which LAs are assigned to their most likely class^36^). Comparative tests included *Vuong-Lo–Mendell–Rubin,*^37^ and *parametric bootstrap ratio likelihood* between *k* class and *k-1* class models (where *P* < 0.05 rejects the smaller models). Finally, plots were assessed for residual autocorrelation. The identification of the optimal model was primarily based upon outlined statistics, but supplemented by clinical plausibility (informed by prior analyses^7^). No uniform threshold for the plausibility of models based upon a minimum proportion of LAs within a single class was applied.^38^

As we observed high entropy values, class assignments were adopted as the dependent variables in logistic regression models to test the association with predictor variables. Generalized linear models with binomial logit link function were employed to estimate the likelihood of being assigned to each class based on key socio-demographic predictors.

Sensitivity analyses were undertaken and are detailed in Appendix C.

## Results

Across the 150 LAs unweighted mean obesity prevalence in Reception was 9.93% (2007/08) remaining stable at 9.90% in 2023/24. In Year 6 it increased from 18.94% in 2007/08 to 22.66% by 2023/24. Unweighted mean IMD score was 23.18 in 2010 and 22.97 in 2019, whilst mean average IDACI scores were 23.60 and 17.96. Mean percentage of the population identifying as White decreased from 76.16% in 2009/10 to 67.17% in 2023/24.

For both Reception and Year 6, optimal models separated LAs into two classes each. In both cases the two and three-class models exhibited negligible difference between AIC and adjusted BIC values, but could be distinguished between based upon classification confidence (entropy in excess of minimum) and likelihood ratio tests, which exhibited no improvement in model fit for three-classes (Table 1).

**Table 1:** LGMM fit statistics based upon obesity prevalence data for 150 upper tier local authority area samples (2007/08–2018/19 and 2021/22–2023/24).

	Reception							Year 6					
	1-Class Model		2-Class Model		3-Class Model			1-Class Model		2-Class Model		3-Class Model	
N	150		150		150			150		150		150	
**Model Fit Criteria**													
AIC	6389.5		**6378.6**		6380.0			8347.9		**8322.6**		8319.5	
Adjusted BIC	6388.6		**6377.0**		6377.8			8347.0		**8321.0**		8317.3	
Entropy	1.00		**0.86**		0.78			1.00		**0.93**		0.77	
L-M-R adjusted LRT (*k* – (*k-1*))	N/A		**<0.01**		0.18			N/A		**<0.01**		0.06	
Parametric Bootstrap LRT	N/A		**<0.01**		0.17			N/A		**<0.01**		0.06	
**Class Trajectory Coefficients**													
Class 1: Intercept	9.69	<0.01	**9.27**	**<0.01**	9.37	<0.01		18.00	<0.01	**17.42**	**<0.01**	15.41	<0.01
Class 1: Trend	−0.00	0.98	**0.02**	**0.02**	0.05	<0.01		0.30	<0.01	**0.32**	**<0.01**	0.17	<0.01
Class 2: Intercept			**12.54**	**<0.01**	10.03	<0.01				**23.24**	**<0.01**	18.55	<0.01
Class 2: Trend			**−0.15**	**<0.01**	−0.09	<0.01				**0.12**	**<0.01**	0.40	<0.01
Class 3: Intercept					10.14	<0.01						22.94	<0.01
Class 3: Trend					0.19	<0.01						0.12	<0.01
**Assigned Class**													
	N	%	**N**	**%**	N	%		N	%	**N**	**%**	N	%
Class 1	150	100	**132**	**88**	81	54		150	100	**135**	**90**	50	33.3
Class 2			**18**	**12**	61	41				**15**	**10**	83	55.3
Class 3					8	5						17	11.3

For practical purposes, where fit statistics and comparative tests diminished subsequent *k* + 1 class models were not tested.

Bold values represent the optimal model.

**Figure 1:**
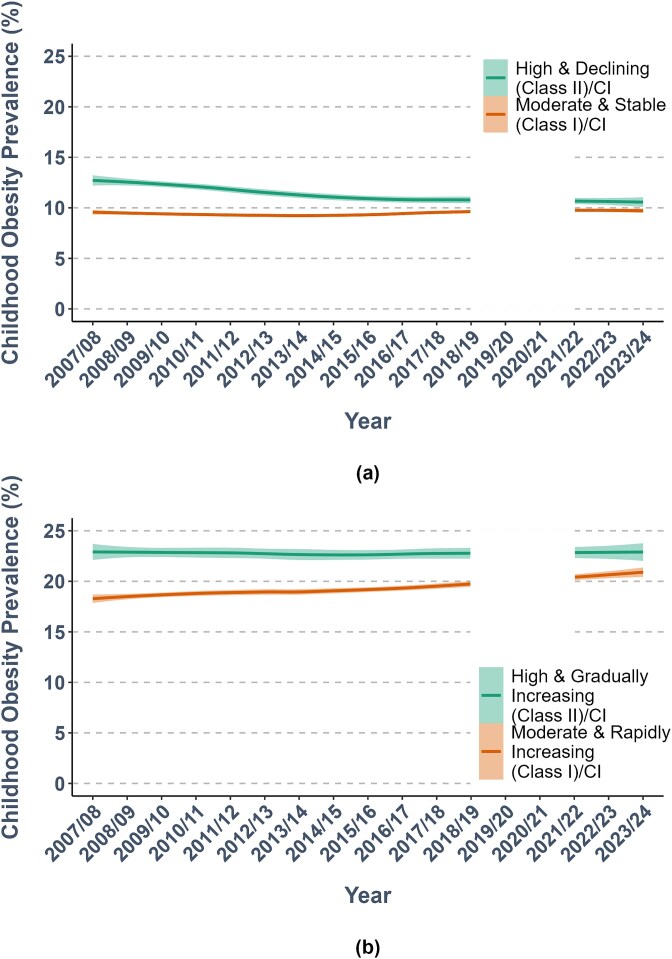
Graphical depiction of assigned obesity prevalence class trajectories. (a) Reception trajectories of obesity (including severe obesity) prevalence; (b) Year 6 trajectories of obesity (including severe obesity) prevalence. Modelled linear trajectories are shown in solid colour lines as Loess smoothed linear trends, with shaded confidence intervals.

For Reception, 88% (*n =* 132) of LAs were assigned to a prevailing ‘Moderate & Stable’ class (Class I) characterized by an initial moderate prevalence of obesity [9.27 (*SE 0.14, P < 0.01*)] that did not change significantly over time [0.02 (*SE < 0.01, P 0.02*)]. The other 12% (*n =* 18) were assigned to a divergent ‘High & Declining’ class (Class II), characterized by higher initial prevalence [12.54 (*SE 0.36, P < 0.01*)], which declined over time [−0.15 (*SE 0.02, P < 0.01*)]. For Year 6, 90% (*n =* 135) were assigned to a prevailing ‘Moderate & Rapidly Increasing’ class (Class I); with initial moderate prevalence [17.42 (*SE 0.22, P < 0.01*)] preceding a statistically significant increasing rate [0.32 (*SE 0.01, P < 0.01*)]. Around 10% of LAs (n = 15) were assigned to a divergent ‘High & Gradually Increasing’ class (Class II), characterized by a higher prevalence [23.24 (*SE 0.53, P < 0.01*)] and more gradual increase over time [0.12 (*SE 0.04, P < 0.01*)] (Fig. 1).

**Figure 2:**
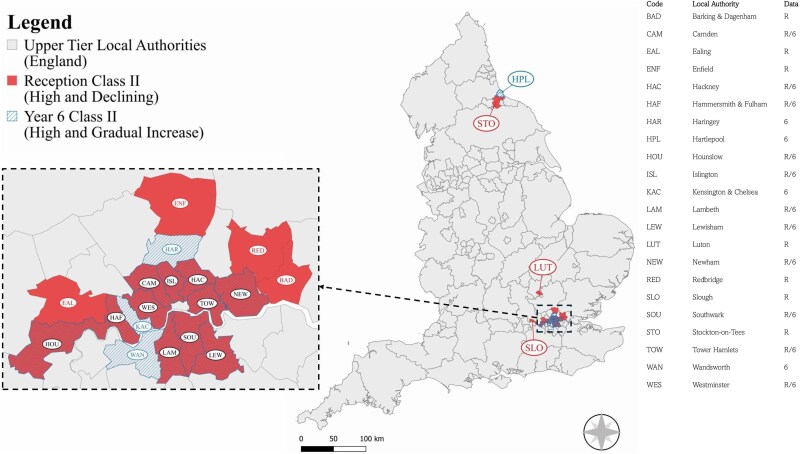
Map depicting geographic locations of class II (reception and year 6) upper tier local authorities in England.

Of those LAs assigned to Class II the majority were in London and the South-East (Reception, 17/18, 94%; Year 6, 14/15, 93%). The two authorities outside London and the South-East, were both neighbours within the North-East. There was significant geographic overlap between Reception and Year 6 Class II; eleven authorities identified as Class II for both, all in London (Fig. 2).

In both instances, higher deprivation (IMD and IDACI for 2010 and 2015, but only for IDACI for 2019) and a higher proportion of children from ethnic minority groups were associated with Class II membership in Reception and Year 6 (Table 2). Keeping all other variables constant, for every one unit increase in average IMD score the odds of being assigned to Class II increased by 10% [1.10 (*1.04–1.18*)] for Reception and 13% [1.13 (*1.06–1.23*)] for Year 6 (2010); and 9% [1.09 (*1.02–1.17*)] for Reception and 10% [1.10 (*1.03–1.19*)] for Year 6 (2015). For every 1% increase in the proportion of children living in families identified as being income deprived (IDACI score), the odds of being assigned to Class II increased by 22% [1.22 (*1.13–1.34*)] in Reception and 23% [1.23 (*1.14–1.37*)] in Year 6 (2010); 20% [1.20 (1.10–1.33)] in Reception and 23% [1.23 (*1.11–1.38*)] in Year 6 (2015); and 10% [1.10 (*1.01–1.20*)] in Reception and 11% [1.11 (1.02–1.23)] in Year 6 (2019). At each time point for every 1% increase in the proportion of children identifying as White, the odds of being assigned to Class II (Reception or Year 6) were between 8% [0.92 (*0.89–0.96*)] and 11% [0.89 (0.84–0.93)] lower (for detailed associations refer to Table 2).

**Table 2:** Associations obtained using unadjusted logistic regression between classification of reception and year 6 obesity trajectory classes II and hypothesized predictors in 2010, 2015, 2019, and 2024 (where available) (panel **A**). Associations obtained using unadjusted logistic regression between assignment to reception and year 6 obesity trajectory classes II and the relative improvement in IMD and IDACI rank between 2010 and 2019; and change in proportion of ethnic minority group between 2009/10 and 2023/24 (panel **B**).

A	Reception			
Predictor	Odds Ratio 2010	Odds Ratio 2015	Odds Ratio 2019	Odds Ratio 2024
Indices of Multiple Deprivation (IMD)	1.10 (1.04–1.18)	1.09 (1.02–1.17)	1.04 (0.98–1.10)	
Income Deprivation Affecting Children Index (IDACI)	1.22 (1.13–1.34)	1.20 (1.10–1.33)	1.10 (1.01–1.20)	
Proportion White Ethnic Group^a^	0.90 (0.85–0.93)	0.89 (0.84–0.93)	0.89 (0.84–0.93)	0.89 (0.84–0.93)
Proportion ‘Black’ Ethnic Minority Group^a^	1.17 (1.11–1.25)	1.17 (1.11–1.26)	1.20 (1.12–1.30)	1.22 (1.13–1.34)
Proportion ‘South Asian’ Ethnic Minority Group^a^	1.08 (1.04–1.13)	1.08 (1.04–1.12)	1.08 (1.04–1.12)	1.07 (1.03–1.11)
Proportion ‘Other Asian’ Ethnic Minority Group^a^	1.22 (1.04–1.47)	1.17 (1.01–1.38)	1.21 (1.03–1.44)	1.12 (0.94–1.31)
Proportion ‘Other’ Ethnic Minority Group^a^	1.27 (1.15–1.42)	1.26 (1.14–1.40)	1.25 (1.14–1.39)	1.22 (1.13–1.34)
Proportion Unclassified Ethnicity^a^	1.64 (0.67–3.69)	3.07 (1.24–7.65)	3.97 (1.83–9.19)	2.58 (1.54–4.59)
	**Year 6**			
Indices of Multiple Deprivation (IMD)	1.13 (1.06–1.23)	1.10 (1.03–1.19)	1.04 (0.97–1.11)	
Income Deprivation Affecting Children Index (IDACI)	1.23 (1.14–1.37)	1.23 (1.11–1.38)	1.11 (1.02–1.23)	
Proportion White Ethnic Group^a^	0.91 (0.87–0.94)	0.92 (0.88–0.95)	0.92 (0.88–0.95)	0.92 (0.89–0.96)
Proportion Black Ethnic Minority Group^a^	1.19 (1.12–1.28)	1.18 (1.11–1.27)	1.21 (1.13–1.33)	1.24 (1.14–1.38)
Proportion South Asian Ethnic Minority Group^a^	1.04 (1.00–1.08)	1.04 (1.00–1.08)	1.03 (0.99–1.07)	1.02 (0.98–1.06)
Proportion Other Asian Ethnic Minority Group^a^	1.12 (0.93–1.33)	1.10 (0.93–1.29)	1.13 (0.93–1.33)	1.05 (0.85–1.25)
Proportion Other Ethnic Minority Group^a^	1.41 (1.24–1.67)	1.36 (1.21–1.59)	1.36 (1.21–1.58)	1.32 (1.19–1.51)
Proportion Unclassified Ethnicity^a^	3.10 (1.33–8.17)	4.44 (1.73–12.00)	5.88 (2.52–15.51)	3.58 (2.01–7.10)
B	Reception		Year 6	
Change Predictor	Class II Odds Ratio		Class II Odds Ratio	
Inverse change in relative IMD rank (2010–2019)	1.09 (1.05–1.14)		1.12 (1.08–1.18)	
Inverse change in relative IDACI rank (2010–2019)	1.06 (1.04–1.09)		1.05 (1.03–1.08)	
Percentage point change proportion White Ethnic Group (2009/10–2023/24)	1.22 (1.11–1.36)		1.55 (1.32–1.95)	
Percentage point change proportion Black Ethnic Minority Group (2009/10–2023/24)	0.70 (0.60–0.79)		0.68 (0.58–0.78)	
Percentage point change proportion South Asian Ethnic Minority Group (2009/10–2023/24)	1.05 (0.90–1.20)		0.55 (0.38–0.76)	
Percentage point change proportion Other Asian Ethnic Minority Group (2009/10–2023/24)	0.53 (0.31–0.84)		0.66 (0.40–1.05)	
Percentage point change proportion Other Ethnic Minority Group (2009/10–2023/24)	1.42 (1.07–1.93)		1.42 (1.04–1.96)	
Percentage point change proportion Unclassified Ethnicity (2009/10–2023/24)	3.63 (1.82–7.69)		3.91 (1.88–8.66)	

a Reference group = all other ethnic groups.

The odds of assignment to either Class II were also associated with changes in deprivation and ethnicity. For every 1 rank relative improvement in IMD between 2010 and 2019, the odds of assignment to Class II increased by 9% [1.09 (*1.05–1.14*)] for Reception and 12% (1.12 (*1.08–1.18*)) for Year 6. Whilst for every 1 relative rank improvement in IDACI, odds of assignment to Class II increased by 6% [1.06 (*1.04–1.09*)] and 5% [1.05 (*1.03–1.08*)] respectively for Reception and Year 6. Increased proportions of the primary school population identifying as White, Other ethnic minority groups and Unclassified between 2010 and 2024 were universally associated with increased odds of assignment to either Class II. Whilst increased proportions of Black ethnic minority groups were associated with decreased odds of assignment to Class II. Increased proportions of South Asian and Other Asian ethnic minority children were associated with decreased odds of assignment to Class II in Year 6 and Reception, respectively (Table 2).

## Discussion

### Main finding of this study

Our research identified two groups with different trends in obesity prevalence in both Reception and Year 6 children. For most local authorities, prevalences were ‘Moderate & Stable’ for Reception and ‘Moderate & Rapidly Increasing’ for Year 6. A minority, predominantly located in London and the South-East exhibited less adverse trends. Although absolute prevalences remained high, these authorities, followed a declining trend in Reception and a gradually increasing trend in Year 6, contrasting with the majority trends (i.e. the rate of increase was either slower or negative such that the gap to the majority narrowed over the research period). These authorities were characterized by high deprivation and larger ethnic minority populations. Over time, relative deprivation in these areas declined, accompanied by demographic shifts in the child population.

### What is already known on this topic?

Evidence around local authority outliers in childhood obesity trends in England is limited. Public Health England previously identified lower tier local authorities with more favourable z-BMI trends (2006/07–2014/15), after adjusting for deprivation, ethnicity and urban/rural status. Subsequent work identified local activities that might account for such better trends. Factors highlighted, included linkage between weight management services and schools; the adoption of ‘whole school’ approaches; and a focus upon early years nutrition and physical activity.^39^

Similarly, Leeds, reported significantly better trends (2009–2017) than national comparators. It was postulated that this could be due to the introduction of a weight management programme in more deprived areas of the city.^22^ Although subsequent data suggests the favourable trend did not persist.^3^

Mirroring our findings, a latent trajectory analysis of 2007/08 to 2015/16 NCMP data identified two-classes in Reception, but three in Year 6. These broadly aligned with our classes, although Year 6 included an additional class with relatively low and stable prevalence. Assignment to each outlier class was associated with midpoint deprivation and two-category ethnicity.^7^

Whilst many countries have reported increases in childhood obesity during the period of our study, and some have produced sub-national estimates, we are not aware of any that have explicitly examined subnational variation in trends over time. Reductions in childhood obesity in Amsterdam, in contrast to increasing trends in the Netherlands as a whole, were observed in the 2010s. It has been postulated that population change or city led polices could explain the changes.^19^^,^^40^ A variety of factors may contribute towards differences in obesity, including culturally patterned dietary and physical activity behaviours, structural inequalities, and differential environmental exposure.^41^ Such can influence the level and trajectory of obesity risk over time, particularly in areas with changing demographic and deprivation profiles.

### What this study adds

Our study builds on and extends previous work by covering a longer time-period and demonstrating associations with *changes* in deprivation and ethnicity. Additionally, we highlight the geographic clustering of ‘outliers’ (both with respect to overlap between Reception and Year 6 outliers; and their spatial proximity), which we suggest strengthens the differences being systematic rather than statistical noise. The extension of data beyond the COVID-19 pandemic suggests that trajectory differences between groups persisted and may reflect a return to longer-term patterns as data normalized.^42^ Whilst group differences may appear modest, small percentage point shifts translate to large absolute numbers in high-prevalence areas.

We think two mechanisms warrant further explanation, particularly given the persistence of the findings over time. First the changes may reflect population changes: changes in ethnic make-up or the socio-economic status of families with children. In support of this, London is an area of high population churn and some of the descriptive data shows changes in population and deprivation indices.^43^ However other cities and other parts of London may also be susceptible to similar forces, but were not identified as showing the same pattern; and it is unclear if the observed changes are of the nature, scale and duration to explain the observed changes.

Second, the patterns may be accounted for by a set of policies, activities or investment delivered across parts of London and/or the wider South-East. These may be specific to obesity or may be more general areas of government activity. For example, London had a childhood obesity plan and has adopted measures like breakfast clubs, universal free school meals and restrictions on advertising of less-healthy food on its public transport network; investment in infrastructure leading to urban regeneration; or higher levels of investment (or greater protection during a time of austerity) for public services (either directly or by support from the third sector).^44–46^ The wider geographic reach may suggest regional rather than LA factors, although networks of practice may facilitate the sharing of learning and good practice between LAs. However, it is not immediately clear why some of these London wide influences might play out differently in some areas compared to others.

Despite these differences, it’s important to note that ⁓90% of local authorities followed similar trajectories, despite marked variation in deprivation and ethnicity. Moreover, whilst we highlight some relatively favourable trends, overall trends are concerning, particularly for Year 6 where prevalence is higher than in Reception and continues to rise.

### Limitations of this study

As an observational study our approach is not able to establish causal explanations. As with similar work, our analysis should be seen as the starting point to explore what may be driving the patterns.^20^

Sex stratified data at local authority level are not published, we were therefore unable to consider differential trajectories between sexes.^47^ Although, sex specific differential impacts at the local-level may be unlikely.^7^ Data is also unavailable with sufficient temporality at lower geographic scale, meaning that we are unable to explore intra-authority health inequalities.^48^

Class assignment based upon LGMMs is subject to uncertainty based upon model parameter constraints and the complexity of response variable.^49^ The class trajectories we identified were similar to previous analysis,^7^ whilst our modelling approach allowed for random effects and converged despite its small sample, indicating adequate capture of individual variability and long-term trends.^38^

Despite strong model fit, use of a large comprehensive national surveillance dataset and extensive sensitivity analyses to test the robustness of findings, analyses may be subject to measurement error based upon inconsistencies in data collection practices.^50^

We employed unadjusted logistic regression to estimate associations between predictors and class membership, which may underestimate the strength of associations.^34^ A bias-adjusted sensitivity analysis was conducted, and the associations remained consistent. Moreover, our models were constrained to those with high entropy (>80% for most classes) mitigating against this effect.^35^

As the indices used to measure deprivation change between time points, we were not able to estimate differences in scores over time, but instead identified changes in relative deprivation rank for LA.^31^ As such it is difficult to establish the extent to which an individual LA has undergone absolute change, only the extent to which it has changed relative to other areas.

Ethnicity data was based upon the entire primary school population, whilst NCMP data reflect only specific year groups. Despite this inconsistency the broader child-specific measure we used over a longer period enhanced the granularity of our analysis compared to prior studies.^7^

## Conclusions

Our findings identify a statistically distinct groups of LAs, primarily located in the South-East of England, with diverging trajectories for childhood obesity prevalence for both Reception and Year 6. Many of these authorities are neighbours and share similar socio-demographic characteristics. Whilst the findings may reflect changes in the population or a proactive approach to policy making and investment in public realm and services, the study does not establish causation. Given the high prevalence of childhood obesity, these relatively small changes may yield valuable improvements in child population health, and they warrant further investigation to identify whether these trends are driven by local policy initiatives, broader regional factors, or shifts in population composition.

## Supplementary Material

Appendix_A-B_Summary_Table_fdaf103

Final_proofed_Appendix_C_fdaf103

## Data Availability

Summary data tables are available *online* as supplementary materials (Appendices A and B), and code can be shared upon request.

## Notes

**Dr. John Rahilly (PhD)** is a Research Fellow in Public Health Data Science and Quantitative Methods at the UCL Great Ormond Street Institute of Child Health. His research interests are in the application of quantitative methods to analyze childhood obesity.

**Prof. Mario Cortina-Borja (PhD)** is a Professor of Biostatistics at the UCL Great Ormond Street Institute of Child Health. He is an applied statistician with an interest in epidemiology, seasonal patterns, statistical methods and modelling; and has published over 490 peer-reviewed papers.

**Dr. Oliver Mytton (PhD)** is a Clinical Associate Professor and Honorary Public Health Consultant at the UCL Great Ormond Street Institute of Child Health. His research interests are in population-level and policy interventions to improve dietary behaviours or increase physical activity, with particular focuses on children and local authorities.
