# Supplementary material for: Trends in childhood obesity for upper tier local authorities in England between 2007/08 and 2023/24: a latent trajectory analysis
Source: J Public Health (Oxf). 2025 Aug 19;47(4):e668–77. doi: 10.1093/pubmed/fdaf103 (PMC12669991; doi:10.1093/pubmed/fdaf103)
Supplement: Final_proofed_Appendix_C_fdaf103 [file final_proofed_appendix_c_fdaf103.docx]

**Appendix C: Sensitivity Analyses**

We undertook the following sensitivity analyses to test whether identified models and derived associations were robust. Such can broadly be split into those that tested the robustness of the LGMM model and those that were used to test associations with predictors (primarily changes in predictor variables over time).

First, LGMMs were fitted against overweight and obesity data (≥ 85^th^ Centile) from the NCMP for the primary research period (2007/08 to 2023/24; excluding 2019/20 and 2020/21). Based upon Reception data the inclusion of overweight resulted in the optimal model being identified as a single-class, suggesting no strong evidence for distinct groups within the population. This group was characterised by a high initial prevalence (23%), but statistically significant gradually declining trend over time (-0.01) (table A1). The identification of a single group, characterised by a declining trend suggested there was neither an increase in overweight and obesity nor any statistically distinguishable outliers characterised by divergent trends. For Year 6 data, a comparable two-class model was identified as optimal, in which the groups were distinct based upon a small outlier class (18/150) estimated to have a higher initial prevalence (38.25, where the majority group was 31.87), but divergent non-significant trend over time (0.04, where the majority group was 0.29). This could suggest that similar effects to those originally identified persisted when we accounted for overweight and obesity in Year 6 (**table A1**).

| **Table A1**: LGMM fit statistics based upon overweight and obesity (≥ 85^th^ Centile) prevalence data for 150 Upper Tier Local Authority area samples (2007/08 – 2018/19 and 2021/22 – 2023/24). | | | | | | | | | | | | | |
| --- | --- | --- | --- | --- | --- | --- | --- | --- | --- | --- | --- | --- | --- |
|  | **Reception** | | | | | |  | **Year 6** | | | | | |
|  | **1-Class Model** | | 2-Class Model | | 3-Class Model | |  | 1-Class Model | | **2-Class Model** | | 3-Class Model | |
| N | **150** | | 150 | | 150 | |  | 150 | | **150** | | 150 | |
| **Model Fit Criteria** | | | | | | | | | | | | | |
| AIC | **8706.2** | | 8709.5 | | 8704.0 | |  | 9029.9 | | **9010.6** | | 9010.9 | |
| Adjusted BIC | **8705.3** | | 8708.0 | | 8701.8 | |  | 9029.0 | | **9009.0** | | 9008.8 | |
| Entropy | **1.00** | | 0.34 | | 0.63 | |  | 1.00 | | **0.90** | | 0.73 | |
| L-M-R adjusted LRT (*k* – (*k-1*)) | **NA** | | 0.35 | | 0.01 | |  | NA | | **<0.01** | | 0.12 | |
| Parametric Bootstrap LRT | **NA** | | 0.34 | | <0.01 | |  | NA | | **<0.01** | | 0.10 | |
| **Class Trajectory Coefficients** | | | | | | | | | | | | | |
| Class 1: Intercept | **23.01** | **<0.01** | 24.08 | <0.01 | 23.58 | <0.01 |  | 32.65 | <0.01 | **31.87** | **<0.01** | 33.00 | <0.01 |
| Class 1: Trend | **-0.01** | **<0.01** | -0.16 | <001 | -0.14 | <0.01 |  | 0.26 | <0.01 | **0.29** | **<0.01** | 0.37 | <0.01 |
| Class 2: Intercept |  |  | 22.00 | <0.01 | 22.49 | <0.01 |  |  |  | **38.25** | **<0.01** | 29.47 | <0.01 |
| Class 2: Trend |  |  | 0.04 | 0.47 | 0.09 | 0.11 |  |  |  | **0.04** | **0.42** | 0.11 | 0.01 |
| Class 3: Intercept |  |  |  |  | 16.63 | <0.01 |  |  |  |  |  | 38.15 | <0.01 |
| Class 3: Trend |  |  |  |  | <0.01 | 0.99 |  |  |  |  |  | 0.04 | 0.58 |
| **Assigned Class** | | | | | | | | | | | | | |
|  | **N** | **%** | N | % | N | % |  | N | % | **N** | **%** | N | % |
| Class 1 | **150** | **100** | 78 | 52 | 99 | 66 |  | 150 | 100 | **132** | **88** | 93 | 62 |
| Class 2 |  |  | 72 | 48 | 47 | 31 |  |  |  | **18** | **12** | 40 | 27 |
| Class 3 |  |  |  |  | 4 | 3 |  |  |  |  |  | 17 | 11 |
| * For practical purposes, where fit statistics and comparative tests diminished subsequent *k*+1 class models were not tested. | | | | | | | | | | | | | |

Second, the original LGMMs were rerun over a period including observations originally removed due to reduced sample size during the COVID pandemic (2019/20 – 2020/21). In both instances, optimal models remained two-classes with trends consistent with the primary analysis. For Reception, a small outlier class (27/150) was identified with a high initial prevalence (12.08%), but declining trend (-0.10). In comparison to a majority characterised by lower initial prevalence (9.09%), but increasing trend (0.04). For Year 6, a small group (9/150) were identified with a high initial prevalence (23.00%) and gradually increasing trend (0.12). In comparison to a majority characterised by lower initial prevalence (17.45%), but more rapidly increasing trend (0.30). In both instances there was negligible difference between two and three-class models, with entropy thresholds commonly the factor used to differentiate between them. However, model fit statistics suggest that the analysis was robust to additional data (**table A2**).

| **Table A2**: LGMM fit statistics based upon obesity prevalence data for 150 Upper Tier Local Authority area samples (2007/08 - 2023/24). | | | | | | | | | | | | | |
| --- | --- | --- | --- | --- | --- | --- | --- | --- | --- | --- | --- | --- | --- |
|  | **Reception** | | | | | |  | **Year 6** | | | | | |
|  | 1-Class Model | | **2-Class Model** | | 3-Class Model | |  | 1-Class Model | | **2-Class Model** | | 3-Class Model | |
| N | 150 | | **150** | | 150 | |  | 150 | | **150** | | 150 | |
| **Model Fit Criteria** | | | | | | | | | | | | | |
| AIC | 7404.9 | | **7395.1** | | 7399.1 | |  | 8862.3 | | **8840.2** | | 8834.3 | |
| Adjusted BIC | 7404.0 | | **7393.5** | | 7397.0 | |  | 8861.4 | | **8838.7** | | 8832.1 | |
| Entropy | 1.00 | | **0.81** | | 0.85 | |  | 1.00 | | **0.92** | | 0.76 | |
| L-M-R adjusted LRT (*k* – (*k-1*)) | NA | | **<0.01** | | 0.44 | |  | NA | | **<0.01** | | 0.01 | |
| Parametric Bootstrap LRT | NA | | **<0.01** | | 0.41 | |  | NA | | **<0.01** | | <0.01 | |
| **Class Trajectory Coefficients** | | | | | | | | | | | | | |
| Class 1: Intercept | 9.63 | <0.01 | **9.09** | **<0.01** | 9.09 | <0.01 |  | 18.04 | <0.01 | **17.45** | **<0.01** | 18.68 | <0.01 |
| Class 1: Trend | 0.01 | 0.09 | **0.04** | **<0.01** | 0.03 | <0.01 |  | 0.28 | <0.01 | **0.30** | **<0.01** | 0.38 | <0.01 |
| Class 2: Intercept |  |  | **12.08** | **<0.01** | 12.06 | <0.01 |  |  |  | **23.00** | **<0.01** | 22.70 | <0.01 |
| Class 2: Trend |  |  | **-0.10** | **<0.01** | -0.10 | <0.01 |  |  |  | **0.12** | **<0.01** | 0.12 | <0.01 |
| Class 3: Intercept |  |  |  |  | 9.68 | <0.01 |  |  |  |  |  | 15.39 | <0.01 |
| Class 3: Trend |  |  |  |  | 0.22 | <0.01 |  |  |  |  |  | 0.17 | <0.01 |
| **Assigned Class** | | | | | | | | | | | | | |
|  | N | % | **N** | **%** | N | % |  | N | % | **N** | **%** | N | % |
| Class 1 | 150 | 100 | **123** | **82** | 123 | 82 |  | 150 | 100 | **136** | **91** | 81 | 54 |
| Class 2 |  |  | **27** | **18** | 23 | 15 |  |  |  | **14** | **9** | 17 | 11 |
| Class 3 |  |  |  |  | 4 | 3 |  |  |  |  |  | 52 | 35 |
| * For practical purposes, where fit statistics and comparative tests diminished subsequent *k*+1 class models were not tested. | | | | | | | | | | | | | |

Third, the original LGMMs were rerun with the addition of weights reflecting the size of the sample contributing to the prevalence value as an offset over the complete time-period (2007/08 to 2023/24 inclusive). With sample weights adopted as an offset, optimal models remained largely consistent with the unweighted models, which form the basis of the main analysis. A two-class model was assessed as best fitting regarding Reception data, in which a small group (22/150) was identified as having higher initial prevalence (16.44%) than the majority group (13.48%), but a divergent, downward trend over time (-1.0 compared to 0.04). For Year 6 a three-class model was marginally deemed a better fit, with a resultant small group (15/150), characterised by higher initial prevalence (19.48%) than the majority (13.71%), but less rapidly increasing trend over time (0.11 compared to 0.29). Sample size was estimated to have a statistically significant, but inverse relationship (Reception) with obesity prevalence in all of the models (**table A3**).

| **Table A3**: LGMM fit statistics based upon obesity prevalence data for 150 Upper Tier Local Authority area samples (2007/08 – 2023/24) with sample size added as an offset covariate. | | | | | | | | | | | | | |
| --- | --- | --- | --- | --- | --- | --- | --- | --- | --- | --- | --- | --- | --- |
|  | **Reception** | | | | | |  | **Year 6** | | | | | |
|  | 1-Class Model | | **2-Class Model** | | 3-Class Model | |  | 1-Class Model | | **2-Class Model** | | 3-Class Model | |
| N | 150 | | **150** | | 150 | |  | 150 | | **150** | | 150 | |
| **Model Fit Criteria** | | | | | | | | | | | | | |
| AIC | 7378.9 | | **7374.7** | | 7372.7 | |  | 8860.1 | | **8835.8** | | 8826.7 | |
| Adjusted BIC | 7377.9 | | **7373.0** | | 7370.4 | |  | 8859.1 | | **8834.1** | | 8824.3 | |
| Entropy | 1.00 | | **0.80** | | 0.78 | |  | 1.00 | | **0.93** | | 0.79 | |
| L-M-R adjusted LRT (*k* – (*k-1*)) | NA | | **0.02** | | 0.05 | |  | NA | | **<0.01** | | <0.01 | |
| Parametric Bootstrap LRT | NA | | **0.01** | | 0.05 | |  | NA | | **<0.01** | | <0.01 | |
| **Class Trajectory Coefficients** | | | | | | | | | | | | | |
| Class 1: Intercept | 14.50 | <0.01 | **13.48** | **<0.01** | 14.80 | <0.01 |  | 14.77 | <0.01 | **13.71** | **<0.01** | 14.39 | <0.01 |
| Class 1: Trend | 0.02 | 0.05 | **0.04** | **<0.01** | 0.05 | <0.01 |  | 0.27 | <0.01 | **0.29** | **<0.01** | 0.37 | <0.01 |
| Class 2: Intercept |  |  | **16.44** | **<0.01** | 14.81 | <0.01 |  |  |  | **19.48** | **<0.01** | 18.51 | <0.01 |
| Class 2: Trend |  |  | **-0.10** | **<0.01** | -0.06 | <0.01 |  |  |  | **0.11** | **<0.01** | 0.11 | <0.01 |
| Class 3: Intercept |  |  |  |  | 14.77 | <0.01 |  |  |  |  |  | 10.88 | <0.01 |
| Class 3: Trend |  |  |  |  | 0.18 | <0.01 |  |  |  |  |  | 0.17 | <0.01 |
| log(Sample Size) | -0.61 | <0.01 | **-0.54** | **<0.01** | -0.65 | <0.01 |  | 0.42 | 0.43 | **0.48** | **0.01** | 0.57 | <0.01 |
| **Assigned Class** | | | | | | | | | | | | | |
|  | N | % | **N** | **%** | N | % |  | N | % | **N** | **%** | N | % |
| Class 1 | 150 | 100 | **128** | **85** | 74 | 49 |  | 150 | 100 | **135** | **90** | 78 | 52 |
| Class 2 |  |  | **22** | **15** | 63 | 42 |  |  |  | **15** | **10** | 17 | 11 |
| Class 3 |  |  |  |  | 13 | 9 |  |  |  |  |  | 55 | 37 |
| * For practical purposes, where fit statistics and comparative tests diminished subsequent *k*+1 class models were not tested. | | | | | | | | | | | | | |

Fourth, the original LGMMs were rerun with data limited to 2007/08 to 2018/19 (excluding the period directly affected by the COVID-19 pandemic and all subsequent years). When restricted to the pre-pandemic period, a consistent two-class model was estimated as optimal in regards to Reception data. Consistent with the primary analysis, a small group (14/150), characterised by high initial prevalence (12.91%) and declining trend (-0.22) were identified. This was in contrast to a majority group with lower initial prevalence (9.40%) and non-significant trend. Whilst in Year 6 a three-class model was estimated as optimal, characterised by moderate (18.36%), high (24.09%) and low (15.62%) initial prevalences, with respectively increasing (0.19), non-significant (<0.01) and decreasing (-0.02) trends. Although providing differing model classes the general narrative suggests towards the existence of a minimum of two groups of LAs across Reception and Year 6, which diverge from the majority (**table A4**).

| **Table A4**: LGMM fit statistics based upon obesity prevalence data for 150 Upper Tier Local Authority area samples (2007/08 – 2018/19). | | | | | | | | | | | | | | | |
| --- | --- | --- | --- | --- | --- | --- | --- | --- | --- | --- | --- | --- | --- | --- | --- |
|  | **Reception** | | | | | |  | **Year 6** | | | | | | | |
|  | 1-Class Model | | **2-Class Model** | | 3-Class Model | |  | 1-Class Model | | 2-Class Model | | **3-Class Model** | | 4-Class Model | |
| N | 150 | | **150** | | 150 | |  | 150 | | 150 | | **150** | | 150 | |
| **Model Fit Criteria** | | | | | | | | | | | | | | | |
| AIC | 5118.7 | | **5107.3** | | 5113.2 | |  | 5987.6 | | 5975.2 | | **5972.2** | | 59735 | |
| Adjusted BIC | 5117.8 | | **5105.7** | | 5111.1 | |  | 5986.7 | | 5973.7 | | **5970.0** | | 5970.7 | |
| Entropy | 1.00 | | **0.90** | | 0.64 | |  | 1.00 | | 0.93 | | **0.89** | | 0.90 | |
| L-M-R adjusted LRT (*k* – (*k-1*)) | N/A | | **<0.01** | | 0.74 | |  | N/A | | <0.01 | | **0.03** | | 0.17 | |
| Parametric Bootstrap LRT | N/A | | **<0.01** | | 0.62 | |  | N/A | | <0.01 | | **0.02** | | 0.14 | |
| **Class Trajectory Coefficients** | | | | | | | | | | | | | | | |
| Class 1: Intercept | 9.77 | <0.01 | **9.40** | **<0.01** | 9.79 | <0.01 |  | 18.61 | <0.01 | 18.14 | <0.01 | **18.36** | **<0.01** | 18.35 | <0.01 |
| Class 1: Trend | -0.02 | 0.90 | **0.01** | **0.60** | -0.03 | 0.10 |  | 0.17 | <0.01 | 0.07 | 0.90 | **0.19** | **<0.01** | 0.21 | <0.01 |
| Class 2: Intercept |  |  | **12.91** | **<0.01** | 12.90 | <0.01 |  |  |  | 24.03 | <0.01 | **24.09** | **<0.01** | 23.79 | <0.01 |
| Class 2: Trend |  |  | **-0.22** | **<0.01** | -0.23 | <0.01 |  |  |  | 0.18 | <0.01 | **<0.01** | **0.91** | <0.01 | 0.99 |
| Class 3: Intercept |  |  |  |  | 8.61 | <0.01 |  |  |  |  |  | **15.62** | **<0.01** | 16.51 | <0.01 |
| Class 3: Trend |  |  |  |  | 0.09 | <0.01 |  |  |  |  |  | **-0.02** | **0.64** | -0.30 | <0.01 |
| Class 4: Intercept |  |  |  |  |  |  |  |  |  |  |  |  |  | 15.66 | <0.01 |
| Class 4: Trend |  |  |  |  |  |  |  |  |  |  |  |  |  | -0.02 | 0.51 |
| **Assigned Class** | | | | | | | | | | | | | | | |
|  | N | % | **N** | **%** | N | % |  | N | % | N | % | **N** | **%** | N | % |
| Class 1 | 150 | 100 | **136** | **91** | 98 | 65 |  | 150 | 100 | 138 | 92 | **125** | **83** | 123 | 82 |
| Class 2 |  |  | **14** | **9** | 37 | 25 |  |  |  | 12 | 8 | **12** | **8** | 13 | 9 |
| Class 3 |  |  |  |  | 15 | 10 |  |  |  |  |  | **13** | **9** | 12 | 8 |
| Class 4 |  |  |  |  |  |  |  |  |  |  |  |  |  | 2 | 1 |
| * For practical purposes, where fit statistics and comparative tests diminished subsequent *k*+1 class models were not tested. | | | | | | | | | | | | | | | |

Fifth, for purposes of interpretation within the primary analysis the change in relative deprivation (IMD and IDACI) was inverted so that “improvement” in rank was a positive rather than negative value. Therefore, sensitivity analysis included the change consistent with the scale of original deprivation ranks (i.e. negative values relate to “improving relative rank”, whereas positive values relate to “worsening” relative rank). Consistent with the primary analysis, negative associations were estimated between worsening relative rank and the odds of assignment to Class II in either Reception or Year 6. For every one unit increase (“worsening”) of relative rank in IMD the odds of assignment to Class II decreased by 8% (0.92 [0.88 - 0.95]) in Reception and 11% (0.89 [0.85 - 0.93]) in Year 6. For every 1 unit increase (“worsening”) of relative rank in IDACI the odds of assignment to Class II decreased by 6% (0.94 [0.92 - 0.96]) in Reception and 5% (0.95 [0.93 - 0.97]) in Year 6. The primary analysis calculated the relative rank change based upon the absolute difference between the deprivation indicator (IMD or IDACI) in 2010 and 2019. An alternative analysis was undertaken in which this change in rank accounted for all three time points (2010, 2015, 2019) through the mean relative changes in rank over time. Associations with this mean change were consistent with the absolute change in rank between 2010 and 2019 throughout. For every one relative rank “improvement” in IMD the odds of assignment to Class II increased by 19% (1.19 [1.11 - 1.29]) in Reception and 26% (1.26 [1.16 - 1.40]) in Year 6. Whilst for every one relative rank “improvement” in IDACI the odds of assignment to Class II increased by 13% (1.13 [1.08 - 1.19]) in Reception and 11% (1.11 [1.06 - 1.17]) in Year 6. Further, changes in deprivation over time were conceptualised as binary variables representing a distinction between LAs that were in the quintiles, quartiles and deciles that had evidenced the greatest relative improvement in rank (in excess of 20 places for both IMD and IDACI) between 2010 and 2019 and those that had not. The identification of an LA as being within the “most improved” quintile or quartile was consistently associated with increased odds of assignment to Class II in either Reception or Year 6. Whilst this association was no longer statistically significant based upon deciles this is likely related to the sample size (**table A5**).

| **Table A5:** Associations obtained using unadjusted logistic regression between assignment to Reception or Year 6 obesity trajectory Class II and alternative predictors of change in deprivation (worsening relative IMD and IDACI rank; mean change in relative IMD and IDACI rank over time; and identification of the LA as being in the upper quintile, quartile and decile based upon relative improvement in IMD and IDACI rank). | | |
| --- | --- | --- |
| **A** | | |
|  | **Reception** | **Year 6** |
|  | **Class II Odds Ratio** | **Class II Odds Ratio** |
| Raw change in relative IMD rank  (2010 – 2019) | 0.92 (0.88 - 0.95) | 0.89 (0.85 - 0.93) |
| Raw change in relative IDACI rank  (2010 – 2019) | 0.94 (0.92 - 0.96) | 0.95 (0.93 - 0.97) |
| Mean raw change in relative IMD rank  (2010, 2015 and 2019) | 0.84 (0.77 - 0.9) | 0.79 (0.72 - 0.86) |
| Mean inverse change in relative IMD rank  (2010, 2015 and 2019) | 1.19 (1.11 - 1.29) | 1.26 (1.16 - 1.40) |
| Mean raw change in relative IDACI rank  (2010, 2015 and 2019) | 0.89 (0.84 - 0.93) | 0.9 (0.86 - 0.94) |
| Mean inverse change in relative IDACI rank  (2010, 2015 and 2019) | 1.13 (1.08 - 1.19) | 1.11 (1.06 - 1.17) |
| Upper quartile relative IMD rank change  (2010 – 2019) | 13.36 (3.84 - 48.84) | 49.12 (12.58 - 231.86) |
| Upper quartile relative IDACI rank change  (2010 – 2019) | 14.38 (4.87 - 45.69) | 12.00 (3.82 - 40.55) |
| Upper quintile relative IMD rank change  (2010 – 2019) | 12.70 (3.37 - 50.44) | 28.66 (7.23 - 131.04) |
| Upper quintile relative IDACI rank change  (2010 – 2019) | 16.16 (4.47 - 63.37) | 9.14 (2.35 - 34.38) |
| Upper decile relative IMD rank change  (2010 – 2019) | 2.53 (0.12 - 21.06) | 3.14 (0.15 - 26.5) |
| Upper decile relative IDACI rank change  (2010 – 2019) | 8.60 (1.48 - 50.27) | 1.86 (0.09 - 12.65) |

Sixth, within the main analysis for purposes of interpretability ethnic minority groups were analysed as raw population percentages, therefore all logistic regression models are relative to all other ethnic groups. Additionally, proportions of each ethnic minority group relative to a White reference were also included as a further sensitivity. For the static variables (2009/10 to 2023/24) regression coefficients were largely consistent with the primary analysis, with higher proportions of the primary school population identifying as being from non-White ethnic minority groups associated with assignment to Class II in both Reception and Year 6 (**table A6: A**). Associations with changes to the proportions of the school population from defined groups are more complex. Mirroring the primary analysis, an increase in the proportion of the population identifying as Black ethnic minority is consistently negatively associated with odds of assignment to Class II in either Reception or Year 6. Similarly, an increase in the proportion of the primary school population identifying as South Asian ethnic minority group is associated with a slight increase in the odds ratio of being assigned to Class II in Reception, consistent with the primary analysis, although slightly weaker in the sensitivity. Where the primary analysis estimated negative associations between an increasing proportion of the population identifying as Other Asian ethnic minority group and assignment to Class II in Reception or Year 6, where based upon a White reference group no clear association existed in Reception and the strength was reduced in Year 6. Similarly in relation to Other Ethnic minority groups, the sensitivity analysis reduced the strength of association in regards to Year 6 and neutralised it in regards to Reception. The change in the proportion of the school population not providing any ethnicity information remained strongly positively associated with increased odds of assignment to Class II in both Reception and Year 6 (**table A6: B**). The outlined inconsistencies between the sensitivity and primary analyses could be attributable to increased sensitivity to changes in the reference population leading to unstable odds ratios or greater statistical noise.

| **Table A6**: Associations obtained using unadjusted logistic regression between classification of Reception and Year 6 obesity trajectory Classes II and hypothesised ethnicity predictors in 2010, 2015, 2019 and 2024 [where available] (Panel **A**). Associations obtained using unadjusted logistic regression between assignment to Reception and Year 6 obesity trajectory Classes II and the relative change in proportion of ethnic minority group (in relation to a White reference) between 2009/10 and 2023/24 (Panel **B**). | | | | |
| --- | --- | --- | --- | --- |
| **A** | **Reception** | | | |
| **Predictor** | **Odds Ratio 2010** | **Odds Ratio 2015** | **Odds Ratio 2019** | **Odds Ratio 2024** |
| Proportion Black Ethnic Minority Group | 1.13 (1.09 - 1.19) | 1.14 (1.09 - 1.21) | 1.16 (1.1 - 1.23) | 1.22 (1.13 - 1.34) |
| Proportion South Asian Ethnic Minority Group | 1.08 (1.05 - 1.11) | 1.07 (1.04 - 1.11) | 1.07 (1.04 - 1.1) | 1.07 (1.03 - 1.11) |
| Proportion Other Asian Ethnic Minority Group | 1.16 (1.08 - 1.27) | 1.16 (1.08 - 1.26) | 1.19 (1.09 - 1.3) | 1.12 (0.94 - 1.31) |
| Proportion Other Ethnic Minority Group | 1.17 (1.11 - 1.26) | 1.19 (1.12 - 1.28) | 1.19 (1.13 - 1.3) | 1.22 (1.13 - 1.34) |
| Proportion Unclassified Ethnicity | 2.42 (1.54 - 4.21) | 3.64 (2.22 - 6.62) | 3.10 (2.10 - 4.99) | 2.58 (1.54 - 4.59) |
|  | **Year 6** | | | |
| Proportion Black Ethnic Minority Group | 1.14 (1.09 - 1.21) | 1.13 (1.09 - 1.20) | 1.15 (1.10 - 1.23) | 1.24 (1.14 - 1.38) |
| Proportion South Asian Ethnic Minority Group | 1.05 (1.02 - 1.08) | 1.04 (1.01 - 1.07) | 1.03 (1.01 - 1.06) | 1.02 (0.98 - 1.06) |
| Proportion Other Asian Ethnic Minority Group | 1.10 (1.03 - 1.19) | 1.10 (1.02 - 1.18) | 1.11 (1.03 - 1.20) | 1.05 (0.85 - 1.25) |
| Proportion Other Ethnic Minority Group | 1.21 (1.13 - 1.33) | 1.20 (1.13 - 1.32) | 1.20 (1.12 - 1.31) | 1.32 (1.19 - 1.51) |
| Proportion Unclassified Ethnicity | 4.49 (2.37 - 9.97) | 4.01 (2.35 - 7.80) | 3.03 (2.04 – 5.00) | 3.58 (2.01 - 7.10) |
| **B** | **Reception** | | **Year 6** | |
| **Change Predictor** | **Class II Odds Ratio** | | **Class II Odds Ratio** | |
| Percentage point change proportion Black Ethnic Minority Group (2009/10 – 2023/24) | 0.82 (0.79 – 0.89) | | 0.77 (0.68 – 0.84) | |
| Percentage point change proportion South Asian Ethnic Minority Group (2009/10 – 2023/24) | 1.01 (0.91 – 1.10) | | 0.61 (0.44 – 0.78) | |
| Percentage point change proportion Other Asian Ethnic Minority Group | 1.00 (0.82 – 1.20) | | 0.89 (0.71 – 1.10) | |
| Percentage point change proportion Other Ethnic Minority Group | 1.17 (1.04 – 1.32) | | 0.99 (0.84 – 1.14) | |
| Percentage point change proportion Unclassified Ethnicity | 3.63 (1.82 – 7.69) | | 3.91 (1.88 – 8.66) | |
| Reference group = White | | | | |

Finally, as unadjusted logistic regression can be considered liable to underestimate the strength of associations with predictors, a bias adjusted model was additionally estimated in which posterior probabilities of assignment to a class were adjusted to account for classification error. Derived results were broadly consistent with the primary unadjusted analyses and support the estimation of associations between higher deprivation and higher proportions of the primary school population identifying as being from ethnic minority groups with assignment to Class II. The consistency with the primary analysis can also be considered to suggest towards the robustness of all primary analyses, potentially attributable to the high entropy in the optimal models (**table A7**).

| **Table A7**: Associations obtained using unadjusted logistic regression between classification of Reception and Year 6 obesity trajectory Classes II and hypothesised predictors in 2010, 2015, 2019 and 2024 [where available]. | | | | |
| --- | --- | --- | --- | --- |
|  | **Reception** | | | |
| **Predictor** | **Odds Ratio 2010** | **Odds Ratio 2015** | **Odds Ratio 2019** | **Odds Ratio 2024** |
| Indices of Multiple Deprivation (IMD) | 1.13 (1.04 - 1.23) | 1.11 (1.02 - 1.21) | 1.05 (0.98 - 1.13) |  |
| Income Deprivation Affecting Children Index (IDACI) | 1.33 (1.10 - 1.61) | 1.26 (1.09 - 1.46) | 1.12 (1.01 - 1.25) |  |
| Proportion White Ethnic Group* | 0.73 (0.69 - 0.76) | 0.75 (0.60 - 0.98) | 0.73 (0.58 – 0.99) | 0.74 (0.70 - 0.77) |
| Proportion Black Ethnic Minority Group* | 1.39 (1.01 - 1.77) | 1.35 (1.03 - 1.88) | 1.36 (1.05 - 1.75) | 1.36 (1.05 - 1.75) |
| Proportion South Asian Ethnic Minority Group* | 1.09 (1.03 - 1.15) | 1.08 (1.03 - 1.14) | 1.08 (1.03 - 1.13) | 1.07 (1.02 - 1.12) |
| Proportion Other Asian Ethnic Minority Group* | 1.97 (0.60 - 6.48) | 1.63 (0.78 - 3.41) | 1.56 (0.89 - 2.73) | 1.51 (0.84 - 2.80) |
| Proportion Other Ethnic Minority Group* | 1.59 (1.18 - 2.15) | 1.54 (1.16 - 2.03) | 1.47 (1.16 - 1.86) | 1.33 (1.10 - 1.81) |
| Proportion Unclassified Ethnicity* | 1.88 (0.60 - 5.93) | 3.98 (1.13 - 14.08) | 4.78 (1.59 - 14.41) | 2.72 (1.21 – 12.78) |
|  | **Year 6** | | | |
| Indices of Multiple Deprivation (IMD) | 1.14 (1.04 - 1.25) | 1.11 (1.02 - 1.20) | 1.05 (0.97 - 1.13) |  |
| Income Deprivation Affecting Children Index (IDACI) | 1.24 (1.10 - 1.41) | 1.26 (1.09 - 1.45) | 1.13 (1.01 - 1.26) |  |
| Proportion White Ethnic Group* | 0.75 (0.52 - 0.96) | 0.84 (0.70 - 1.00) | 0.87 (0.69 - 1.03) | 0.69 (0.54 - 0.92) |
| Proportion Black Ethnic Minority Group* | 1.35 (1.05 - 1.72) | 1.19 (1.13 - 1.25) | 1.18 (1.13 - 1.24) | 1.18 (1.13 - 1.25) |
| Proportion South Asian Ethnic Minority Group* | 1.08 (1.03 - 1.13) | 1.97 (0.60 - 6.48) | 1.63 (0.78 - 3.41) | 1.58 (0.71 - 3.23) |
| Proportion Other Asian Ethnic Minority Group* | 1.56 (0.89 - 2.73) | 1.59 (1.18 - 2.15) | 1.54 (1.16 - 2.03) | 1.58 (1.16 - 2.13) |
| Proportion Other Ethnic Minority Group* | 1.53 (1.22 - 1.92) | 1.47 (1.16 - 1.86) | 1.88 (0.60 - 5.93) | 1.34 (1.19 - 1.50) |
| Proportion Unclassified Ethnicity* | 4.16 (1.11 – 7.14) | 3.98 (1.13 - 14.08) | 4.78 (1.59 - 14.41) | 3.79 (1.82 – 13.02) |
| * Reference group = all other ethnic groups | | | | |
